# Microstructural Evolution of SK85 Pearlitic Steel Deformed by Heavy Cold Rolling

**DOI:** 10.3390/ma15238405

**Published:** 2022-11-25

**Authors:** Cai-Ding Yang, Ye Liu, Gao-Yang Zhou, Xing-Li Zou, Xiong-Gang Lu, Guang-Hui Cao

**Affiliations:** 1 Baosteel Research Institute, Baoshan Iron & Steel Co., Ltd., Shanghai 201900, China; 2 State Key Laboratory of Advanced Special Steel, School of Materials Science and Engineering, Shanghai University, Shanghai 200444, China

**Keywords:** pearlitic steel, cold rolling, microstructure, EBSD, mechanical property

## Abstract

The microstructural evolution of SK85 pearlitic steel cold-rolled up to a 90% rolling reduction was characterized by scanning electron microscopy with electron backscattered diffraction (EBSD) and X-ray diffraction (XRD). SK85 steel exhibits excellent cold rolling performance. The interlamellar spacing of pearlite is refined obviously and a tensile strength of 2318 MPa can be reached for SK85 steel after 90% rolling reduction, an increase of 83% from 1264 MPa before rolling. The EBSD observation indicates that the {001} <110> texture becomes pronounced at a 90% rolling reduction in cold-rolled Sk85 steel. A propagation and multiplication of dislocations occur during rolling as the kernel average misorientation (KAM) angles significantly increase from 0.72° to 2.11°. The XRD analysis reveals that bcc ferrite is transformed into a bct structure at a 90% rolling reduction. The strengthening mechanism was discussed.

## 1. Introduction

Possessing high strength, sufficient ductility, and great strain-hardening behavior achieved by heavy plastic deformation at ambient temperature, pearlitic steel has a wide application in automotive coil springs, suspension bridge cables, and tire cords [1]. The heavy cold rolling of pearlitic steel is expected to broaden the application of high carbon steels to high strength steel sheets [2]. Furthermore, the functional alloys of magnetostriction, Fe-Co alloys, could be prepared by cold-rolling [3]. Pearlite, normally consisting of lamellar ferrite and cementite, is a eutectic decomposition of austenite during isothermal or continuous cooling processes [4,5], which results in high strength without the addition of many strengthening alloying elements such as Nb, Ti, and V. The near eutectoid composition of pearlitic steels can exhibit high strength at a modest cost. These advantages have promoted studies of pearlitic steel focused on the relationship between microstructure and strength after the plastic deformation process. Cold-drawn pearlitic steels are the most representative material due to their ultra-high strength and extra strengthening by deformation-induced cementite decomposition [6,7]. It was observed that pearlite was refined with decreasing interlamellar spacing (ILS) during cold drawing. The ILS of a few nanometers of pearlite can be obtained from 100 nm before cold-drawing [8,9,10,11,12], which is postulated as an effective pathway for mechanical property improvement. The cementite lamellae could act as obstacles for dislocation gliding, and dislocations were mostly present at the interface between ferrite and cementite. The dislocation density increased by three orders of magnitude at a drawing strain of 3.7, revealing that dislocation is the main strengthening mechanism [13]. Apart from the refined pearlite with dislocations at high density, cementite suffers from a partial decomposition in cold-deformed Fe-4.5 at.% C alloy [14]. A pearlitic steel containing 0.7 wt.% of carbon subjected to a strain of 3.5 shows that the cementite phase undergoes a partial dissolution during cold wire drawing [15]. Taniyama et al. [16] reported that the unit cell of ferrite in a drawn pearlitic steel containing 0.9 wt.% C transformed from a bcc to bct structure due to the supersaturation of carbon in ferrite at a true strain larger than 1.5. The decomposition of cementite investigated by atom probe tomography showed that the carbon concentration is about 20 at.% at the ferrite-cementite interface at a drawing strain of 3.5 [17]. The carbon concentration is even further reduced to 12–18 at.% in cementite and the average carbon concentration in ferrite could increase to 0.63 at.% at a drawing strain of 5. The decomposition saturated at a strain large than 5 [18]. Most carbon atoms segregate at the sub-grain boundary in ferrite, suggesting that the dislocation density in ferrite is probably the mechanism underlying cementite decomposition during heavy cold drawing [19]. The strain-induced decomposition of cementite in cold-drawn pearlitic steel also has an important impact on its mechanical property; carbon atoms separated from the cementite were believed to form a Cottrell atmosphere, thereby hindering the movement of the dislocation [20]. As the carbon content in ferrite increases, solid solution hardening is another strengthening factor in cold-drawn pearlitic steel [21]. It is well known that cold-rolling could improve tensile properties. During heavy plastic deformation the crystallography of pearlite changes tremendously. The interlamellar spacing and the misorientation along and across the ferrite lamellae show significant through-diameter variations in wires drawn to large strains [22]. EBSD is an electron diffraction technique in scanning electron microscopy (SEM) uniquely suited to characterizing the crystallographic phase, crystal orientation, and defect density. The KAM derived from the EBSD data is a measure of local grain misorientation [23], which can be used to assess the distribution of local plastic strain and the dislocation density.

So far, investigations into the crystallographic characteristics of cold-rolled pearlitic steels quantified by EBSD are still limited. Most of the previous research has focused on drawn pearlitic steels. Heavy cold rolling of pearlitic steel broadens the industrial engineering applications of high carbon steels [2]. However, the microstructural evolution during heavy cold rolling of pearlitic steels is not yet fully understood. This paper deals with XRD and EBSD analyses of SK85 pearlitic steel at different cold rolling reductions with particular emphasis on the effect of the microstructure and crystallography on the mechanical property. The implication of the present study is expected to be helpful in understanding the mechanical behavior of heavily cold-rolled pearlitic steel and the underlying strengthening mechanism.

## 2. Materials and Methods

The SK85 pearlitic steel sheets used in the present research were provided by Baoshan Iron & Steel Co., Ltd. (Shanghai, China), and were cold rolled from a thickness of 1.8 to 0.18 mm with reduction ratios from 10, 30, 50, 70, to 90%, respectively. The chemical composition of the SK85 steel is shown in Table 1. The microstructure of specimens after rolling was observed by a field-emission JSM-7001F SEM (JEOL, Tokyo, Japan). Before characterization, the specimen surface was ground with waterproof abrasive papers progressively from 400 to 2000 grit, then etched with a 4 vol% nital solution. The crystallographic features of ferrite in the cold-rolled SK85 steel were analyzed using a SU70 Hitachi SEM (HITACHI, Tokyo, Japan) equipped with an EBSD detector. The surface of the as-rolled specimen was polished for 4 h in a Buehler VibroMet 2 vibratory polisher (Buehler, Lake Bluff, Illinois, USA) with a 0.04 μm colloidal silica slurry after a standard grinding procedure. The EBSD scan was performed with a step size of 50 nm at an operating voltage of 15 kV. The KAM represents the average numerical grain misorientation where the maximum misorientation angle is 5° [24,25,26,27]. The raw data were analyzed using TSL-OIM analysis software (7.0 version). For the XRD analysis the longitudinal cross-sections of the as-rolled pieces were mechanically polished and then electropolished using 20 vol% HClO4 in glacial acetic acid to eliminate residual stress in the mechanically polished layers. The diffractograms were measured by a D/MAX 2500 diffractometer (Rigaku, Tokyo, Japan) with Cu Kα1 radiation with a wavelength of λ = 0.1541 nm operating at 40 kV and 250 mA with a scanning speed of 0.2°/min from 42.8° to 46.4° and from 80.5° to 84.1°, respectively. To evaluate the mechanical properties of the SK85 steel after different cold rolling reductions, tensile tests were performed under ambient conditions in an MTS C40 (MTS, Eden Prairie, MN, USA) electronic universal testing machine at a strain rate of 2 × 10^−4^ s^−1^. There are various mechanical tests that could be used to determine the properties required in the product specifications. Randjbaran et al. [28] investigated the CNT epoxy composite laminates’ flexural modulus of elasticity and bending toughness at room temperature using the three-point bending test specified in ASTM D790. In this case, the mechanical properties of the SK85 steel were determined in accordance with the standard testing method of ASTM A370-10 [29]. This test method covers procedures and definitions for the mechanical testing of wrought and cast steel, stainless steel, and related alloys.

## 3. Results

**Figure 1:**
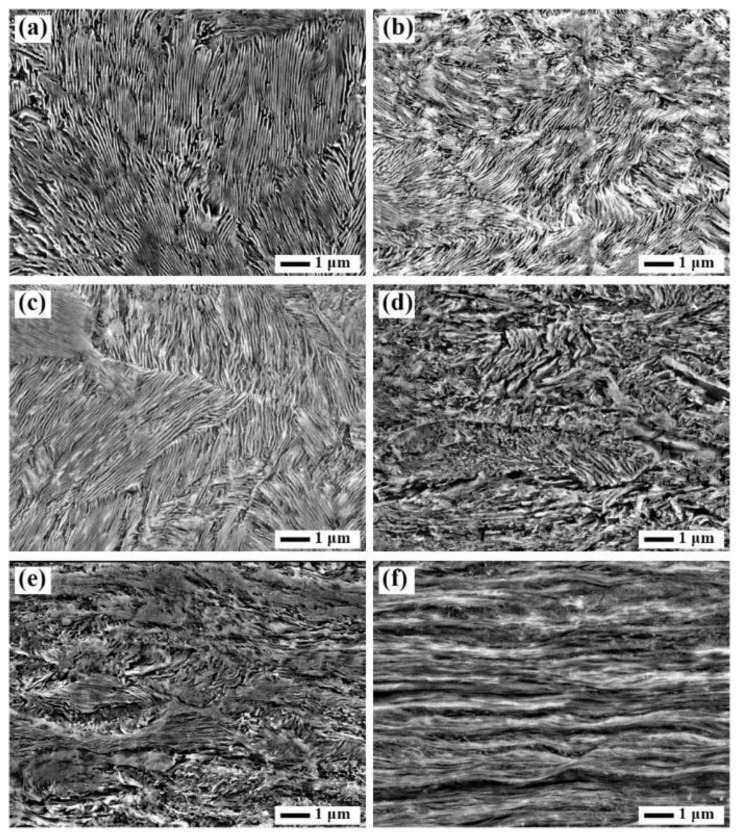
SEM micrographs showing the pearlite structure of the SK85 steel before cold rolling (**a**) and at a rolling reduction of (**b**) 10%; (**c**) 30%; (**d**) 50%; (**e**) 70%; and (**f**) 90%.

Figure 1a is a SEM micrograph indicating the pearlite with an ILS of 120 nm in SK85 steel before rolling. The lamellar pearlite was bent and refined with an ILS of 85 nm at a 10% reduction ratio as shown in Figure 1b. The ILS decreased to about 80 nm at a 30% reduction ratio, and the fracture of the pearlite was hardly observed (Figure 1c). As the rolling reduction increased, the pearlites were extremely bent after a 50% reduction ratio. The cementite lamellae exhibited partial fragmentation (Figure 1d,e) at 50 and 70% reduction ratios leading to difficulty in measuring the ILS. Figure 1f shows that the twisting of cementite is severe and the pearlite is heavily folded at a 90% reduction ratio.

**Figure 2:**
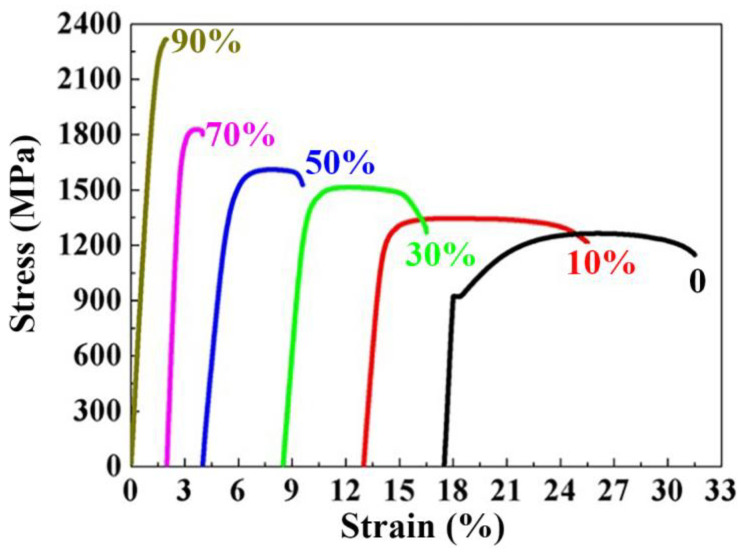
Tensile stress-strain curves of the SK85 steel at different rolling reductions.

The room temperature tensile stress-strain curves of the cold-rolled SK85 at different cold rolling reductions are shown in Figure 2. The SK85 steel shows a distinct yield point before rolling, which was not observed after cold rolling. The yielding strength (YS), ultimate tensile strength (UTS), and fracture strain of the SK85 steel up to a 90% reduction ratio are listed in Table 2. The SK85 steel without deformation has a lower YS (924 MPa) and UTS (1264 MPa). The strength was greatly enhanced, whereas the fracture strain was obviously decreased with increasing rolling reduction ratios. At a 50% reduction ratio the YS and UTS of the SK85 steel increased to 1419 and 1611 MPa, then increased substantially to 2161 and 2318 MPa, respectively, at a 90% reduction ratio. Before cold rolling the SK85 steel exhibited very good ductility with a fracture strain of 14.0%, which was reduced to only 1.8% at a 90% reduction ratio.

**Figure 3:**
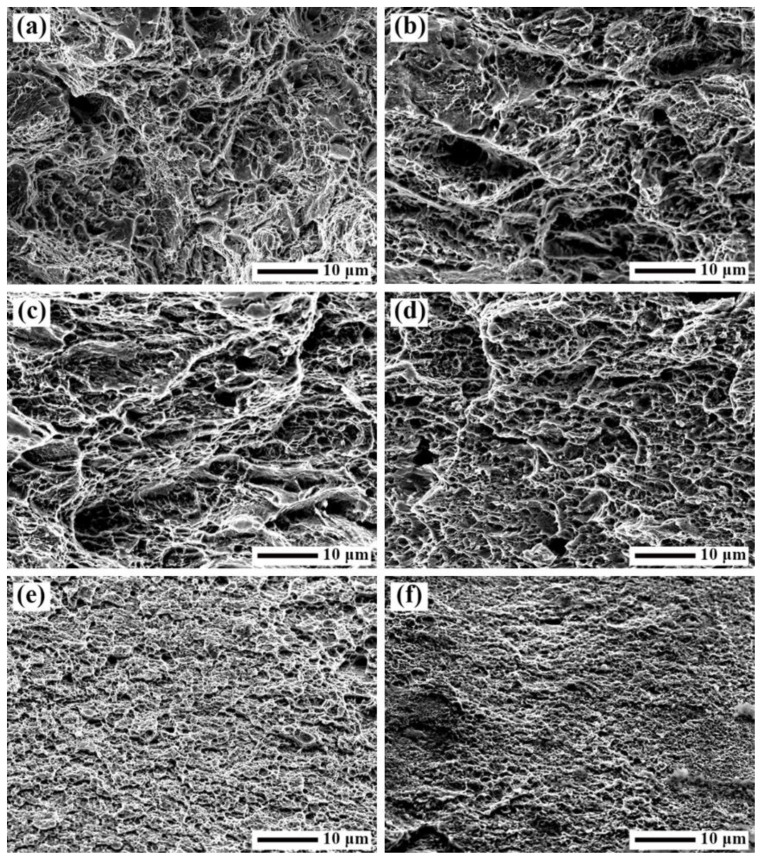
SEM micrographs showing the tensile fracture surfaces of the SK85 steel before cold rolling (**a**) and at a rolling reduction of (**b**) 10%; (**c**) 30%; (**d**) 50%; (**e**) 70%; and (**f**) 90%.

The room temperature tensile fracture surfaces of the SK85 steel at different cold rolling reduction ratios is shown in Figure 3. The fracture surfaces are all composed of dimples, which are characteristic of ductile fracture. A bigger and deeper size of dimples indicates good plasticity. From Figure 3, it can be clearly seen that the size of dimples decreases obviously as the reduction ratio increases, showing that the plasticity of SK85 steel decreases during cold rolling. This conclusion can be drawn from the tensile stress-strain curves of the SK85 steel at different cold rolling reduction ratios shown in Figure 2.

**Figure 4:**
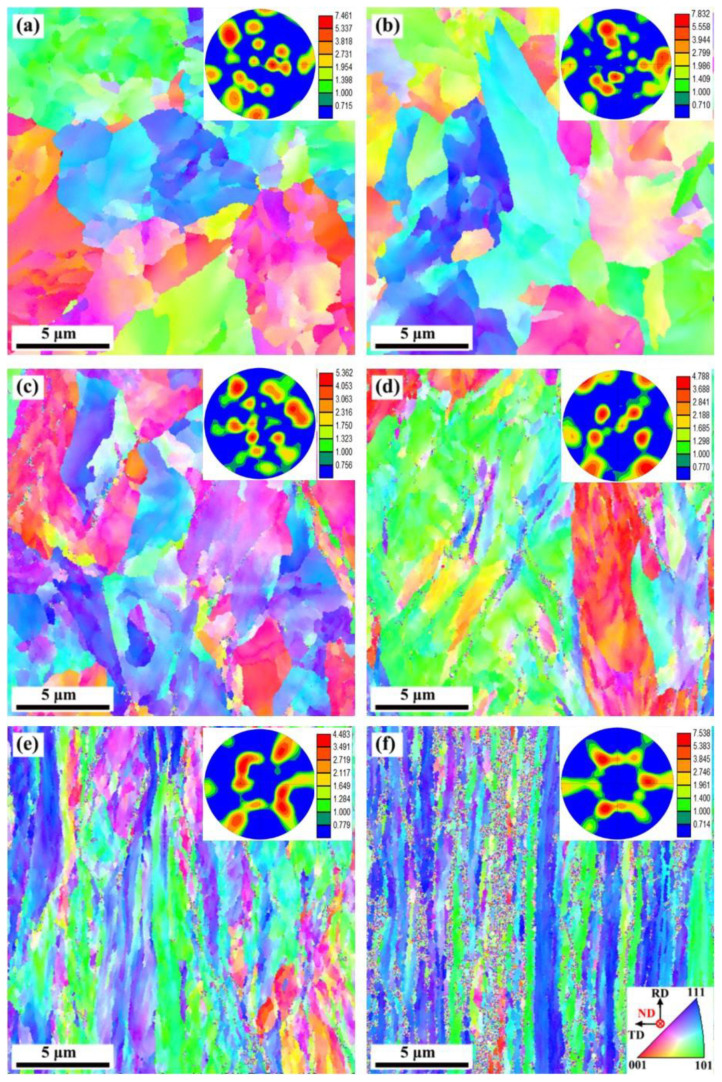
EBSD orientation maps of the rolling direction-transverse direction (RD-TD) planes of the SK85 steel before rolling (**a**) and at a rolling reduction of (**b**) 10%; (**c**) 30%; (**d**) 50%; (**e**) 70%; and (**f**) 90%. The insets are the corresponding {001} pole figures, and {001} is perpendicular to the normal direction (ND).

The EBSD orientation maps of the rolling direction-transverse direction (RD-TD) planes of the SK85 steel at different rolling reductions are shown in Figure 4. The insets are the corresponding {001} pole figures, and {001} is perpendicular to the normal direction (ND). The blue, red, and green represent the <111>, <001>, and <110> directions, respectively. Before rolling the initial texture is randomized and a strong {001} <110> texture is obtained at a 90% rolling reduction.

**Figure 5:**
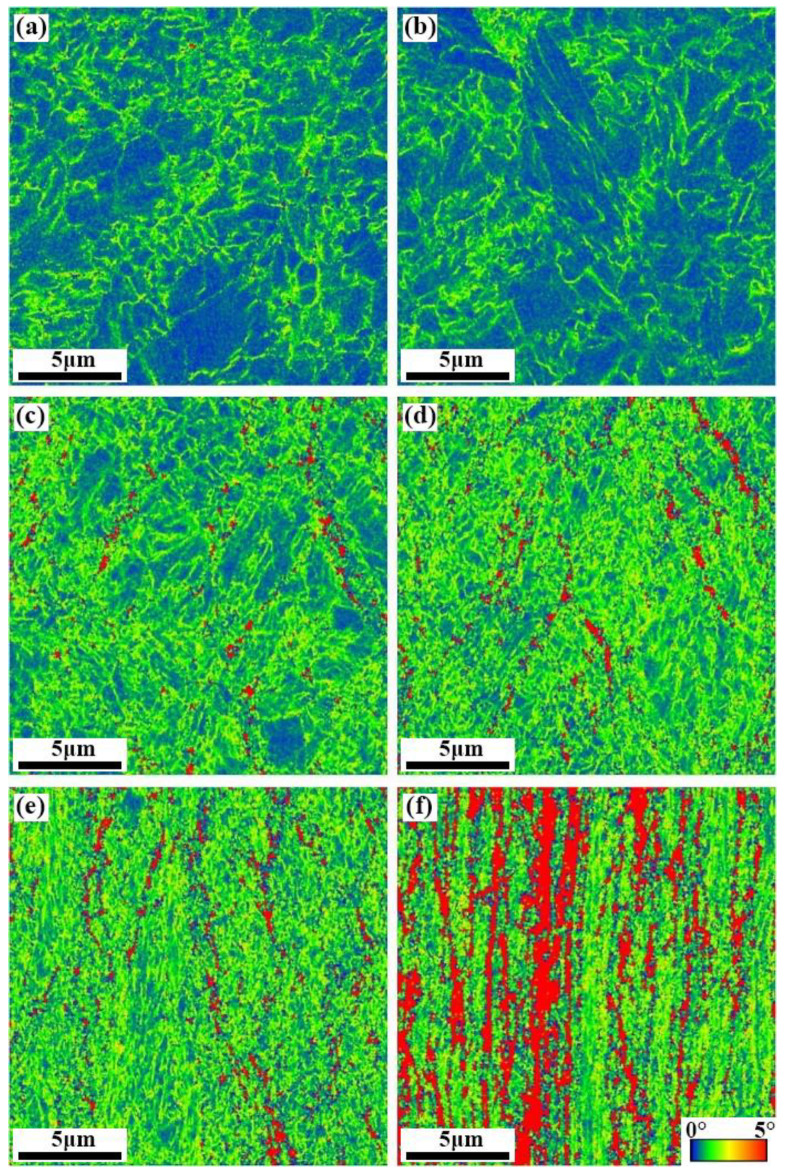
KAM maps of the SK85 steel before rolling (**a**) and at a rolling reduction of (**b**) 10%; (**c**) 30%; (**d**) 50%; (**e**) 70%; and (**f**) 90%.

Figure 5 is the color-coded high-resolution KAM distribution maps of the SK85 steel before and after cold rolling. The bar of blue to red pixels in the KAM maps corresponds to the increasing angles from 0 to 5°. The heterogeneous KAM angles show the deformation induced local orientation gradients inside the ferrites. The larger KAM angles correspond to a higher dislocation density. As clearly shown in the KAM maps, the local plastic strain gradually accumulated and was particularly obvious above a reduction ratio of 70%.

**Figure 6:**
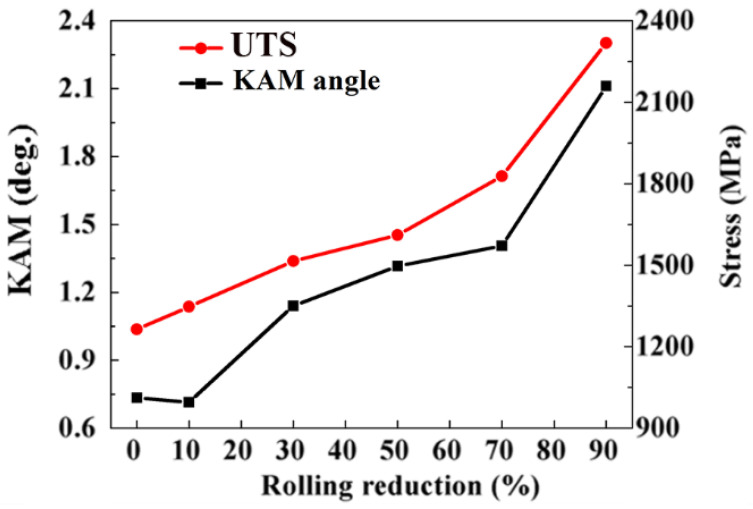
Average KAM angle and UTS of the cold-rolled SK85 steel at various rolling reductions.

The KAM and UTS versus rolling reduction are shown in Figure 6. The average KAM angle of the SK85 steel was about 0.74° at a 10% rolling reduction, which is the same level as in the undeformed specimen (0.72°), suggesting that the accumulation of local plastic strain was not severe in the initial stage of rolling. With the rolling approaching a 70% reduction ratio, the average KAM angle increased to 1.41° indicating dislocation multiplication. SK85 has a KAM as high as 2.11° at a 90% rolling reduction. The UTS of SK85 steel showed an increasing tendency similar to that of KAM, and the higher increasing rates were observed especially above a rolling reduction of 70%.

**Figure 7:**
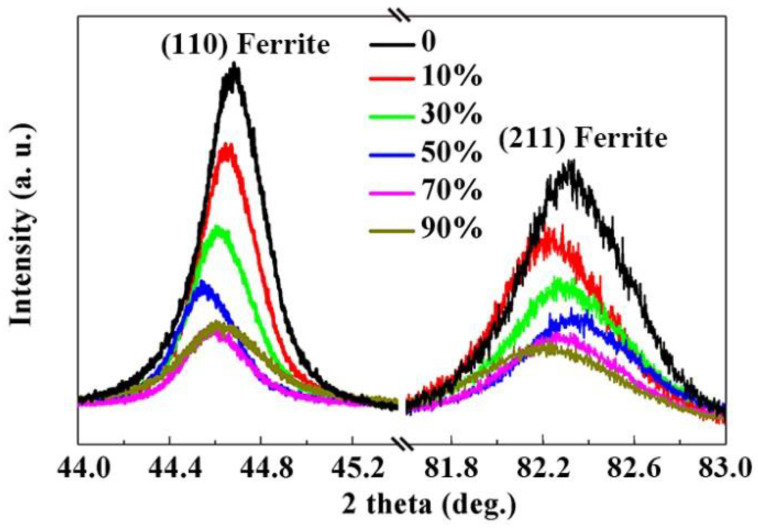
XRD diffractograms of the (110) and (211) ferrite peaks at different rolling reductions.

Figure 7 shows diffractograms of the ferrite (110) and (211) peaks of the cold-rolled SK85 steel. The {110} crystallographic plane is the close packed plane of ferrite. The full width at half maximum (FWHM) of the (110) ferrite peak increased from 0.28° before rolling to 0.31° and 0.42° at 50% and 90% rolling reductions, respectively, displaying the peak broadening effect during cold rolling. The (110) diffraction peaks were remarkably observed to shift to lower angles before the 50% rolling reduction, and then to higher angles. The diffraction angles of the (211) peaks initially decreased at a 10% reduction ratio, became larger with increasing rolling reduction up to 50%, and finally decreased at a 90% rolling reduction.

**Figure 8:**
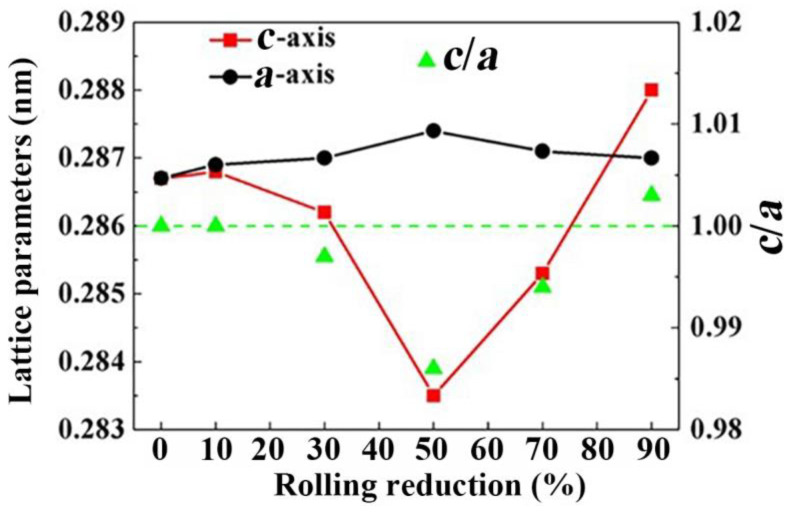
Lattice parameters and *c/a* ratios of ferrites in SK85 steel at different rolling reductions.

The lattice parameters (*a* and *c*) and *c*/*a* ratios of ferrites at different rolling reductions are presented in Figure 8. Before rolling, the ferrite in undeformed SK85 steel had a bcc structure with a lattice parameter *a* of 0.2867 nm and a *c*/*a* ratio of 1. During rolling to 50% the reduction ratio *a* increased slightly to 0.2874 nm while *c* decreased to 0.2835 nm, hence, the *c*/*a* ratio of ferrite decreased from 1 to 0.986. At a rolling reduction over 50% the cementite entered a non-equilibrium state because of the larger rolling strain. As carbon atoms from the decomposing cementite entered ferrites, the *c*/*a* ratio began to increase at a 70% reduction ratio due to the decrease in *a* and the increase in *c*. At 90% rolling reduction the *c*/*a* ratio increased to 1.004 where *a* decreased to 0.2869 nm and *c* increased to 0.2881 nm. When the *c*/*a* ratio of the ferrite exceeded 1 the ferrite showed a bct structure.

## 4. Discussion

The mechanical properties of SK85 steel are greatly dependent on the ILS of pearlite. It is well accepted that cementite lamellae exhibit strong resistance to dislocation glide, which is similar to the effect of grain boundaries in pearlite steels [9,30]. The boundary strengthening effect of decreasing grain size could be caused by two factors: the decreased volume in the formation of dislocation pile-ups against the boundaries, and the increase in resistance to dislocation movement [31,32].

The crystallographic features displayed by the KAM angles indicate the local plastic strain and dislocation development of ferrite in SK85 steel during cold rolling. During plastic deformation, dislocation movement occurs when the resolved shear stress on the slip plane equals the critical resolved shear stress of that slip system [24]. The dislocation propagation and multiplication were not obvious at a 10% rolling reduction (Figure 5 and Figure 6) since the average KAM angle exhibited no large difference compared to the undeformed specimen. Thus, the initial increase in strength at rolling was mostly due to the boundary strengthening resulting from the decrease in ILS. As the rolling reductions increased to 70% and 90%, the average KAM angles dramatically approached 1.42° and 2.11°, revealing that the multiplication of dislocations is dominant. For bcc ferrite the dislocation motion occurs preferentially along the (110) slip plane [27].

The XRD results showed a broadening of the ferrite diffraction peaks. The increased FWHM of (110) ferrite peaks (Figure 7) is another indication of the dislocation density being significantly augmented during cold rolling. Therefore, dislocation strengthening contributed mainly to the high strength of the cold-rolled SK85 steel between 10% and 70% rolling reductions. The {001} <110> texture component become pronounced in the cold-rolled SK85 steel at a 90% rolling reduction. Similar results were obtained in the cold-rolled molybdenum sheets [33,34,35] and a Fe/Ni multilayered composite sheet fabricated by accumulative roll bonding [36]. The presence of {001} <110> texture is considered to increase strength and reduce ductility.

A higher dislocation density is a key factor leading to the strain-induced decomposition of cementite [24,37,38]. The binding energy between the carbon interstitial atom and dislocation in ferrite is 0.75 eV, which is larger than that of carbon atoms in the cementite lattice (0.42 eV). The dislocations were mostly stored in the ferrite lamellae around the ferrite/cementite interfaces during cold rolling [13,38], and their motion could be hindered. Such ferrite dislocations may drag carbon atoms out of cementite into ferrite, resulting in a partial chemical decomposition of the cementite [38]. The bcc to bct transformation and cementite decomposition in the cold-rolled SK85 steel occurred at a 90% rolling reduction where the KAM angle was at its maximum. The strength of the SK85 steel at a 90% rolling reduction can be greatly enhanced by the solid solution hardening related to the decomposition of cementite.

## 5. Conclusions

The microstructural evolution of SK85 pearlitic steel at different rolling reductions has been investigated using XRD and SEM combined with EBSD. The influence of microstructure and crystallography on the mechanical property is investigated. The conclusions can be summarized as follows:

(1) The initial texture is randomized before rolling and a strong {001} <110> texture is obtained at a 90% rolling reduction.
(2) The average KAM angle of ferrite increases from 0.72° before rolling to a maximum of 2.11° at a 90% rolling reduction revealing that dislocation strengthening is the main strengthening mechanism for cold-rolled SK85 steel.
(3) A transition from bcc to bct ferrite occurs at a 90% rolling reduction because of the supersaturation of carbon generated by the strain-induced decomposition of cementite.
(4) The ultimate tensile strength of SK 85 steel is 1264 MPa before rolling, which reaches 2318 MPa at a 90% rolling reduction due to solid solution hardening.

## Figures and Tables

**Table 1:** The chemical composition of the SK85 steel (wt.%).

C	Si	Mn	Cr	Fe
0.86	0.20	0.45	0.25	Bal.

**Table 2:** Mechanical properties of the SK85 steel at different rolling reductions.

Rolling Reduction (%)	0	10	30	50	70	90
**YS (MPa)**	924	1221	1379	1419	1660	2161
**UTS (MPa)**	1264	1347	1515	1611	1828	2318
**Strain (%)**	14.0	12.5	8.0	5.5	2.0	1.8

## Data Availability

Data is contained within the article.
